# CART Peptide Is a Potential Endogenous Antioxidant and Preferentially Localized in Mitochondria

**DOI:** 10.1371/journal.pone.0029343

**Published:** 2012-01-03

**Authors:** Peizhong Mao, Charles K. Meshul, Philippe Thuillier, Natalie R. S. Goldberg, P. Hemachandra Reddy

**Affiliations:** 1 The Division of Neuroscience, Oregon National Primate Research Center, Oregon Health & Science University, Beaverton, Oregon, United States of America; 2 The Department of Public Health & Preventive Medicine, and the Knight Cancer Institute, Oregon Health & Science University, Portland, Oregon, United States of America; 3 The Research Services, Portland VA Medical Center, Portland, Oregon, United States of America; 4 Departments of Behavioral Neuroscience and Pathology, Oregon Health & Science University, Portland, Oregon, United States of America; 5 The Department of Physiology and Pharmacology, Oregon Health & Science University, Portland, Oregon, United States of America; Nathan Kline Institute and New York University School of Medicine, United States of America

## Abstract

The multifunctional neuropeptide Cocaine and Amphetamine Regulated Transcript (CART) is secreted from hypothalamus, pituitary, adrenal gland and pancreas. It also can be found in circulatory system. This feature suggests a general role for CART in different cells. In the present study, we demonstrate that CART protects mitochondrial DNA (mtDNA), cellular proteins and lipids against the oxidative action of hydrogen peroxide, a widely used oxidant. Using cis-parinaric acid as a sensitive reporting probe for peroxidation in membranes, and a lipid-soluble azo initiator of peroxyl radicals, 2,2′-Azobis(2,4-dimethylvaleronitrile) we found that CART is an antioxidant. Furthermore, we found that CART localized to mitochondria in cultured cells and mouse brain neuronal cells. More importantly, pretreatment with CART by systemic injection protects against a mouse oxidative stress model, which mimics the main features of Parkinson's disease. Given the unique molecular structure and biological features of CART, we conclude that CART is an antioxidant peptide (or antioxidant hormone). We further propose that it may have strong therapeutic properties for human diseases in which oxidative stress is strongly involved such as Parkinson's disease.

## Introduction

The neuroendocrine system plays a primary role in the regulation of major physiological processes such as development, growth, metabolism, reproduction, and adaptation to the environment via peptide and steroid hormones, neurotransmitters and their receptors [1]–[4]. The neuropeptide, cocaine- and amphetamine-regulated transcript (CART) gene was first cloned in 1995 [5]. CART has been implicated in a variety of physiological processes, including food intake, reward and endocrine regulations [6], [7]. *CART* knockout (KO) mice have been found to display some aging-associated symptoms, including increased body weight. In humans, a mutation in the *CART* gene has been found in an obese family [8], [9]. Interestingly, a recent report showed that CART levels within cerebrospinal fluid were significantly reduced by 30% in DLB (dementia with Lewy bodies) patients compared to normal controls as well as to Alzheimer's disease patients [10]. The presence of Lewy bodies is considered to be the neuropathologic hallmark of Parkinson's disease (PD), one of the most common neurodegenerative diseases caused by degeneration of dopaminergic neurons in substantia nigra (SN) [11].

In the center nervous system CART is widely expressed in different tissues and regions, including cortex, midbrain and spinal cord [12]–[14]. Increasing studies have shown putative anatomical and functional network between the CART-containing neurons and the mesolimbic dopaminergic system [9], [15]–[18]. The network connecting the SN, nucleus accumbens (NA), and dorsal striatum as well as ventral tegmental area (VTA) is mainly involved in dopaminergic motor pathways, suggesting that CART has the capacity to modulate mesolimbic dopamine, which could have implications for the treatment not only of psychostimulant abuse but also for the treatment of other disorders with midbrain dopamine involvement, such as PD.

Furthermore, CART is highly expressed in the mammalian and human hypothalamus, pituitary and adrenal gland (HPA axis), as well as in the circulatory system [7], [12], [19] (Figure S1). This feature indicates a possible role for CART in stress and natural homeostasis, including the cellular defense system. Interestingly CART expression is regulated by stress in a regionally and time specific manner, and CART is also regulated by corticosteroid actions in the brain, including the hippocampus [9], [20]. Although much is known about CART in terms of its structure, expression and function, little is known about CART receptors and its other interaction proteins, which has hampered basic studies and clinical investigations [7], [21].

Recently using yeast two-hybrid screening, for the first time we identified the mitochondrial protein succinate dehydrogenase, subunit B (SDHB) as the first CART binding partner [22]. SDHB is a critical enzyme for both the Krebs cycle and the mitochondrial electron transport chain (ETC), where it is known as complex II. Furthermore, we found that CART could stimulate the activities of SDH and complex II under basal condition and ischemic condition (oxygen-glucose-deprivation, OGD) and preserve ATP levels after OGD in rat primary neurons [22]. Notably a recent report showed that only one *SDHB* gene mutation caused severe clinical phenotypes in a young patient including multiple system disorders, such as paraganglioma, type 2 diabetes, ischemic stroke, renal failure and congestive heart failure [23]. This further highlights the potential importance of SDHB and CART in pathways compromised in inherited genetic diseases. In addition, a significant common pathological feature of these disorders is oxidative damage. However whether CART has an antioxidant activity is unknown. Thus, in this report we investigated the hypothesis that CART plays a protective role in the oxidative stress using *in vitro* and *in vivo* systems. Interestingly, our data demonstrate that CART peptide is a strong antioxidant and it may be a potential therapeutic candidate for Parkinson's disease.

## Results

### TAT-EGFP-CART protein is an effective CART molecule

In order to increase CART delivery into human cells, we first expressed a CART fusion protein, in which CART is fused with an intracellular transduction vector TAT-EGFP. The plasmid was confirmed by DNA sequencing, and purified TAT-EGFP-CART fusion protein was recognized by a CART-specific antibody using Western blot analysis (Figure S2).

The disulfide bond of many proteins is important for their physiological functions, and it can be chemically altered. To verify that such unique feature of CART peptide is preserved in the CART fusion protein, we treated TAT-EGFP-CART protein and the vector protein TAT-EGFP with or without dithiothreitol (DTT) and labeled all proteins with 4-acetamido-4′-maleimidystilbene-2,2′-disulfonic acid (AMS), then subjected it to an SDS-PAGE. As shown in Figure S3, TAT-EGFP has no significant band shift between proteins treated and untreated with DTT, however TAT-EGFP-CART has an increased molecular weight comparing untreated TAT-EGFP-CART, indicating that the 3 disulfide bonds (6 systeine residues) of CART, the putative CART peptide feature, exist in the CART fusion protein.

Since CART binds to SDHB and stimulates the activity of the SDH/complex II [22], we further tested whether the fusion protein TAT-EGFP-CART retained the properties of CART. As presented in Figure S4, pretreatment of cultured cortical neurons with TAT-EGFP-CART prevents complex II dysfunction after OGD. However, pretreatment with TAT-EGFP protein alone (OGD + vehicle) does not alter complex II activity, suggesting that the effect is specifically linked to CART. Therefore, TAT-EGFP-CART appears to function as effectively as CART peptide alone.

### CART protects cellular mtDNAs, proteins and lipids from oxidative damage

Free radicals yield more extensive damage in mtDNA than in nuclear DNA [24]–[27]. To measure the damage of cellular mtDNA, we first simplified a long template PCR method without using isotopes [25]. DNA lesions resulting from oxidative damage, such as strand breaks and base modifications, block the progression of the polymerase reaction resulting in a decreased amplification of a target sequence. As shown in Supporting Information, the 16.2-kb mitochondrial fragment was successfully amplified in each lane, and H_2_O_2_ treatment reduced the amplification (Figure S5, lanes 3 and 4), especially after 1 h 100 µM treatment, the band density was 22% of normal control. This indicates that mtDNA was significantly damaged following 1 h of H_2_O_2_ treatment. To confirm the PCR products were truly from the mitochondrial genome, we sequenced the PCR products with mitochondrial DNA-specific primers, the results showed that the products were identical to the human mtDNA.

**Figure 1:**
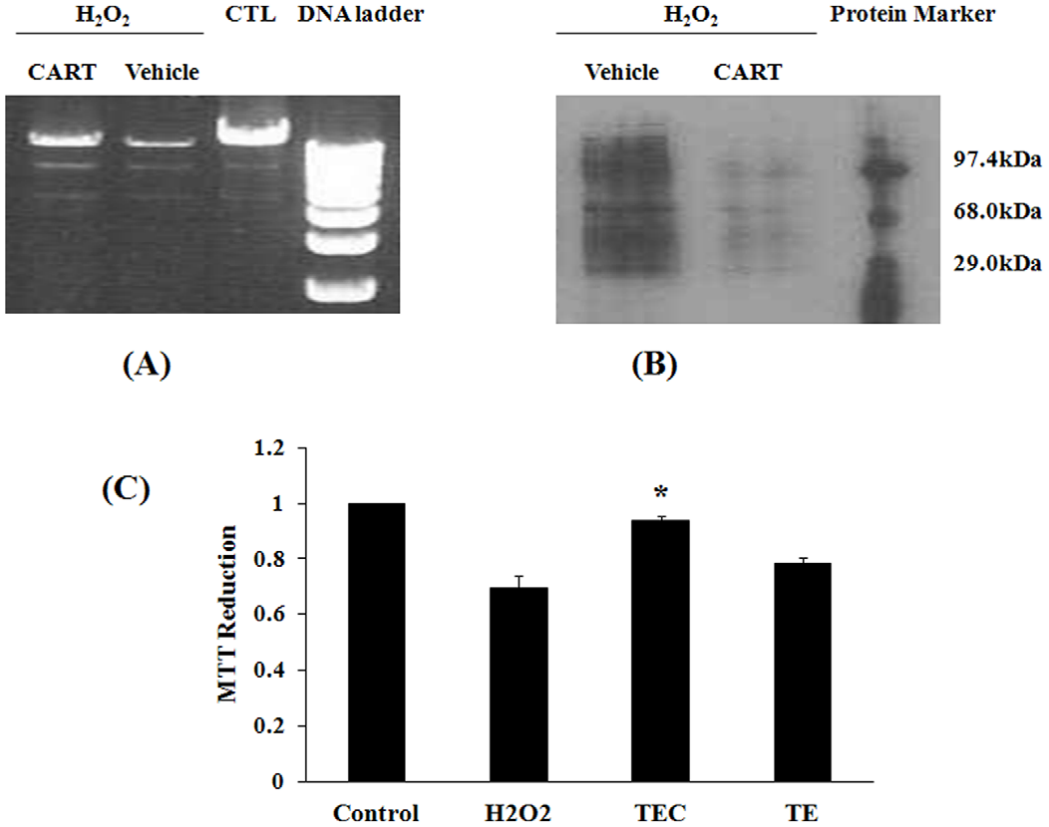
CART reduces oxidative damage in HEK293 cells. (**A**) CART protects mitochondrial DNA damage induced by H_2_O_2_. Total DNA was isolated from HEK293 cells following treatments. Mitochondrial DNAs were amplified by Long PCR. Marker, 1 kb ladder. (**B**) CART reduces hydrogen peroxide-oxidized proteins. Proteins were damaged by reactive oxygen species (H_2_O_2_), vehicle treatment had no effect. However CART fusion protein (TEC 5 µg/ml) decreased the oxidative damage. (**C**) CART diminishes cell death induced by hydrogen peroxide. Cell viability was monitored by mitochondrial function assayed by MTT reduction: H_2_O_2_ (0.1 mM) caused a decline of cellular MTT reduction, and CART treatment, but not vehicle, significantly increased MTT metabolism. *, *P*<0.05, comparing with H_2_O_2_ group, n = 6.

We then evaluated the effect of CART on H_2_O_2_–induced mtDNA damage. H_2_O_2_ treatment combined with TAT-EGFP dramatically reduced mtDNA amplification (Figure 1A, lane H_2_O_2_+Vehicle), showing a similar result with H_2_O_2_ described above (Figure S5). However, pretreatment with the CART fusion protein inhibited the damaging effect of H_2_O_2_ (Figure 1A: lane H_2_O_2_+CART).

To determine the extent of CART protection on oxidative damage we analyzed its effect on protein oxidation by Western blot using the primary antibody against dinitrophenylhydrazine (DNP). As expected, H_2_O_2_ increased protein oxidation (Figure 1B, lane H_2_O_2_+vehicle), pretreatment with TAT-EGFP-CART remarkably reduced protein oxidation (Figure 1B, lane H_2_O_2_+CART).

Because damage to mtDNA was extensive (Figure 1A) we next assessed the physiological state of the mitochondrion in cells treated with H_2_O_2_ and CART. The tetrazolium salt (MTT) method is widely used to measure cell viability. MTT reduction is carried out mainly by mitochondrial SDH, the complex II of the respiratory chain, thus MTT assay reflects SDH activity and mitochondrial function [25]. When HEK293 cells were exposed to H_2_O_2_ for 60 min, a significant decrease in MTT reduction was observed; in other words, H_2_O_2_ treatment induced HEK293 cell death (Figure 1C, bar H_2_O_2_). Therefore, a 60-min exposure to H_2_O_2_ was sufficient to not only induce extensive mtDNA damage, but also to disrupt mitochondrial function and induce cell death. Consistent with its protective effect on mtDNA damage and the complex II assay (Figure S4), TAT-EGFP-CART (Fig. 1C: TEC) provided statistically significant protection against H_2_O_2_ induced cell death (*P*<0.05); however, TAT-EGFP (TE) did not show any significant effect. These data also demonstrate that CART stimulates SDH/complex II activity and preserves mitochondrial function in H_2_O_2_ mediated oxidative stress.

**Figure 2:**
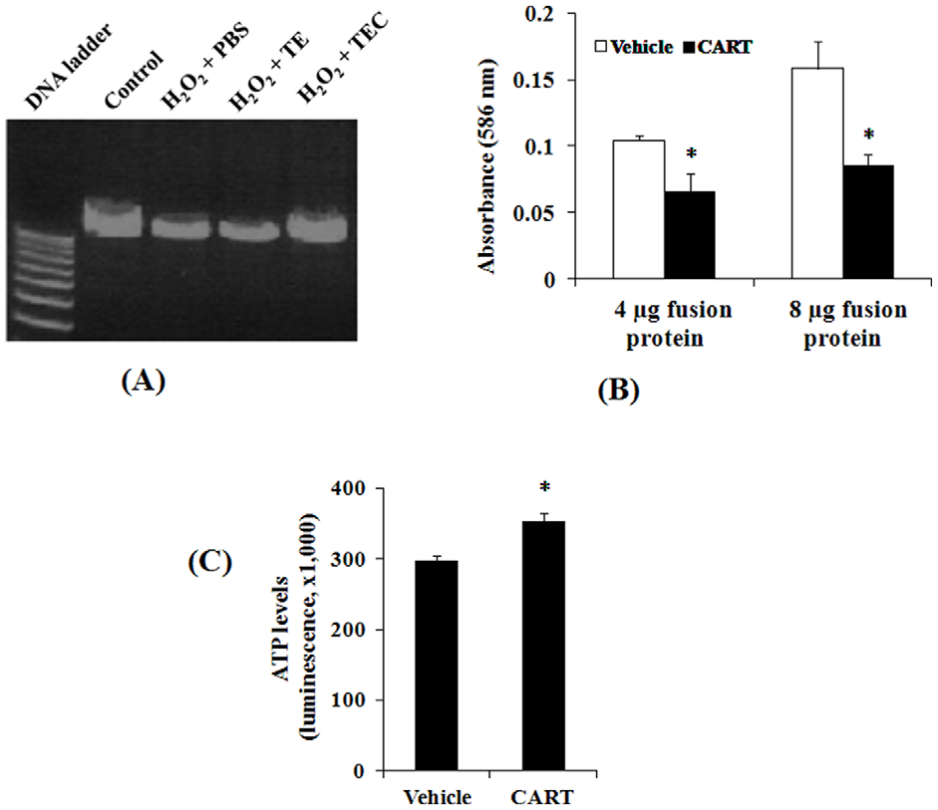
CART antioxidant role in human lymphocytes. (**A**) CART protects mitochondrial DNA damage induced by H_2_O_2_. MtDNAs were amplified by Long PCR as described in Fig. 1. (**B**) CART reduces the levels of lipid peroxidation end products. Protein extracts from lymphocytes treated with 4 and 8 µg TATEGFP-CART were compared to TATEGFP following H_2_O_2_ treatment. Total MDA and HNE formation was detected using 4 mg of total cell homogenates as starting material. Absorbance was measured at 586 nm and data are mean±SEM of 4 independent experiments. *, *P*<0.05, comparing to vehicle group. (**C**) CART preserves cellular ATP production in human lymphocytes. Cultured cells were pretreated with vehicle or CART fusion protein (5μg/ml) and then treated with H_2_O_2_ (0.1 mM) to induce cell death. Cell viability assay performed using a sensitive ATP-based detection method is also an indicator of ATP levels. *, *P*<0.01, comparing to vehicle group, n = 4.

There are increasing correlations between age-related human diseases and oxidative stress processes such as lipid peroxidation. Lipids are the basic components of cell membranes and their oxidative products, especially 4-hydroxyalkenals (4-HNE), initiate structural and functional damage of cells, and increase membrane permeability to ions such as Ca^2+^ [28]. In addition, systemic oxidative stress occurs in some neurodegenerative diseases including PD [29], [30]. Since CART is found in the circulatory system we determined if CART also has an effect on blood cells. Interestingly, the results of mtDNA in HEK cells (Figure 1A) were confirmed by using template DNA isolated from human lymphocytes (Figure 2A) in which CART treatment blocked H_2_O_2_ induced mtDNA damage while no protective effect was seen with TAT-EGFP alone.

To further understand the antioxidant efficacy of CART in human lymphocytes, we measured the accumulation of malondialdehyde (MDA) and 4-HNE as markers of lipid peroxidation. As shown in Figure 2B, H_2_O_2_ induced the generation of MDA and 4-HNE in all groups. Compared to TAT-EGFP (vehicle), total MDA and 4-HNE levels were significantly reduced by TAT-EGFP-CART treatment (CART) for both doses of fusion protein (4 and 8 µg, both *P*<0.05). These data further indicate an anti-oxidative role for CART in human cells.

To understand the change of mitochondrial function in human lymphocytes, we measured cellular ATP levels in cultured lymphocytes by using the CellTiter-Glo Luminescent Assay. Compared to control (TAT-EGFP), TAT-EGFP-CART treatment markedly increases ATP levels (Figure 2C). This assay also faithfully reflects more sensitive ATP-based cell viability suggesting that CART dramatically increases survival of lymphocytes against H_2_O_2_-induced cell damage.

### CART preferentially localized in mitochondria in live HEK cells and cultured primary neurons

Since mitochondria are the main cellular source of oxidants, an antioxidant agent is predicted to be localized to the mitochondria. To test this hypothesis, localization of a TAT-EGFP-CART fusion protein was first examined in live HEK 293 cells using confocal microscopy and MitoTrackor as the mitochondrial indicator. We found extensive colocalization of TAT-EGFP-CART with mitochondria (Figure S6, upper panel). The signal is dispersed and substantially reduced in the control cells (Figure S6, bottom panel). These data are consistent with previous reports indicating that TAT-EGFP could be rapidly transduced into cells and mitochondria, but would not accumulate there [31], addressing a concern about whether CART binding to mitochondrial SDH alters TAT-EGFP affinity with mitochondria. Confocal microscopy revealed that only TAT-EGFP-CART co-localized with MitoTrackor (Figure S6), and TAT-EGFP only transiently and rarely associates.

**Figure 3:**
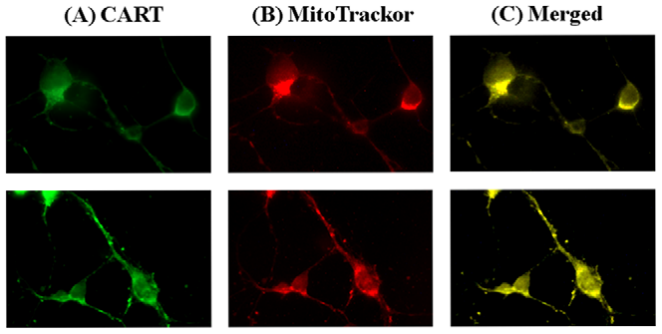
Mitochondrial localization of CART in primary neuronal cells. Mouse cortex neurons (upper panel) and hippocampal neurons (bottom panel) at 7-day in vitro were incubated with MitoTracker Red. The cells were fixed and incubated with a CART specific antibody and secondary antibody, finally treated with tyramide-conjugated fluorescent dye Alexa 488 (green) and photographed using a fluorescence microscope at 100× magnification. (A) shows CART antibody staining (green), (B) shows mitochondrial staining (red), and (c) is merged image (yellow), demonstrating the co-localization of CART and mitochondria.

Similarly, the co-localization of CART and mitochondria was also seen in cultured primary cortex neuronal cells (Figure 3, upper) and hippocampal neuronal cells (Figure 3, lower). Endogenous CART was strongly expressed in neuronal cell body, axon and dendrite, interestingly the mitochondria indicator MitoTracker also strongly detected in the same areas of neuronal cells, demonstrating that CART is localized in mitochondria in cultured neurons.

### CART localized in mitochondrial outer and inner membranes in mouse brain

**Figure 4:**
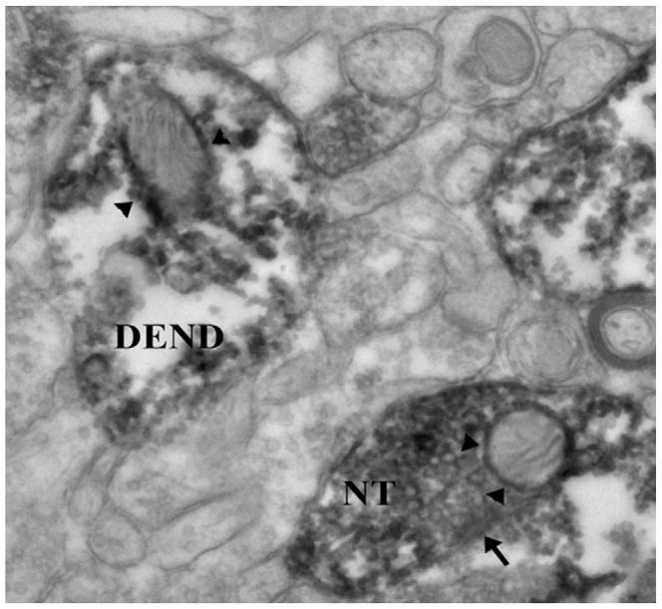
Mitochondrial localization of CART in mouse brain. In the mouse nucleus accumbens determined by electron microscopy. The dark diaminobenzidene reaction product shows that CART is located in a nerve terminal (NT) making a symmetrical synaptic contact (arrow) onto a neuronal cell body. Within the NT is a mitochondrion in which there is CART labeling (arrowheads) around the outer and the inner mitochondrial membranes. Within the photograph is a CART-labeled dendrite (DEND) in which there is a mitochondrion showing CART labeling, particularly along the outer mitochondrion membrane (arrowheads). Magnification: ×40,000.

As mentioned above, CART is highly expressed in nucleus accumbens [7], [14], to further understand the location of native CART in mouse brain, we performed an immunohistochemical study in the nucleus accumbens using electron microscopy. As shown in Figure 4, CART can be found in dendrites, the neuronal cell body, and especially in the nerve terminal (NT). Notably CART labeling is found around the outer mitochondrial membrane. Only in CART-labeled structures are the mitochondria also labeled for CART, showing the specificity of the CART labeling. To our knowledge, this is the first evidence that the CART peptide could be localized to the mitochondria in human cultured cells, mouse primary neurons and mouse brain tissue. This is a novel and important feature of CART peptide as a potential antioxidant since the mitochondrial organelle is the major source of cellular toxic oxidants.

### Direct antioxidant role of CART

CART significantly reduced H_2_O_2_-induced cellular damage as described above. At least partially, this may be due to the interaction between CART and SDH [22]. In addition to this mechanism, and more importantly, CART may directly scavenge reactive oxygen species (ROS).

**Figure 5:**
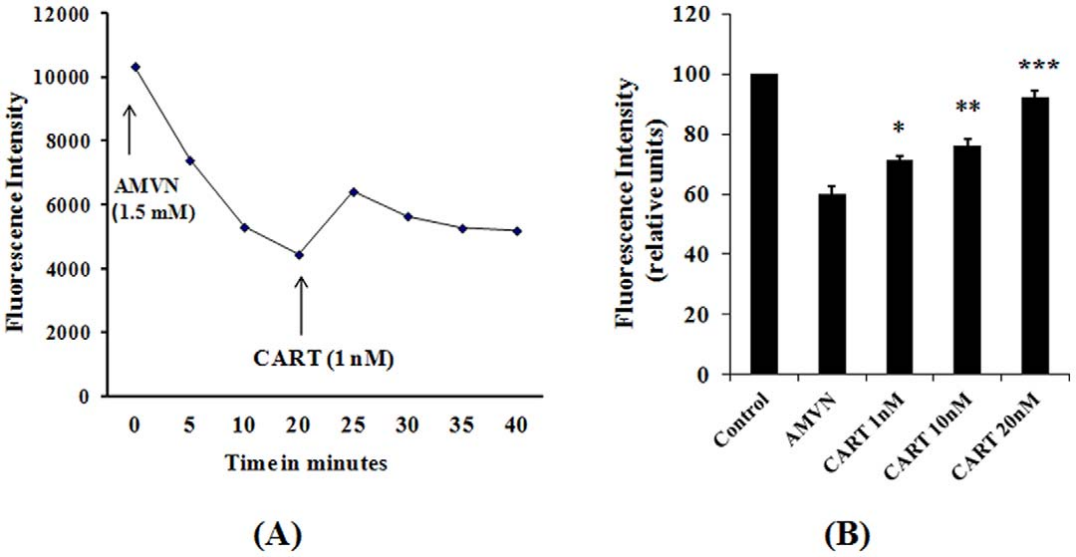
Radical scavenging role of CART. (**A**) Effects of CART on AMVN-induced fluorescence decay in mitochondria isolated from human SHSY cells. AMVN 1.5 mM was added to the reaction medium (0.2 ml) containing mitochondria 50 µg, cis PA 12 µM in 0.1 M KCl and phosphate buffer (50 mM, pH 7.4 at 40°C). CART (1 nM) was added 20 min later. The fluorescence intensity was monitored by using a BIO-TEK spectrofluorometer. (**B**) Radical scavenging activity of CART in mitochondria isolated from HEK293 cells. The reaction containing 50 µg mitochondria, cisPA 6 µM, CART peptides and AMVM 2 mM in 0.1 M KCI and phosphate buffer was performed at 40°C for 20 min, and the fluorescence intensity was measured after continued incubation at RT for 90 min. *, *P*<0.05; **, *P*<0.01; ***, *P*<0.001, comparing with AMVM group.

To investigate the direct antioxidant property of CART, we used a fluorescent polyunsaturated fatty acid, cis-parinaric acid (cisPA) as a sensitive and reliable reporting probe for peroxidation in membranes. Attenuation of its fluorescence is a good index of the oxidative stress at the site where cisPA is present. Thus, we used this assay for testing CART's properties against peroxyl radicals, major oxygen free radicals that damage biomembranes. This method has previously been used to investigate the antioxidant activity of Vitamin E, ubiquinol 10, MitoQ, etc. [32], [33]. As shown in Figure 5A, incubation of mitochondria isolated from human dopaminergic neuroblastoma SH-SY5Y cells with cisPA and 2,2′-Azobis(2,4-dimethylvaleronitrile) (AMVN), a lipid-soluble azo initiator of peroxyl radicals caused cisPA fluorescence decay. Interestingly, 1 nM of CART dramatically reduced the fluorescence decay, demonstrating that CART is an antioxidant. Additionally, the reaction of scavenging oxidants by CART was very fast; we could detect the fluorescence increase within 2 min after addition of the CART peptide. The speed of this effect implies an immediate activity by CART, and suggests that it is not dependent on a signaling cascade of antioxidant gene/protein expression.

Very interestingly, low dose (1 nM) of CART shows an antioxidant activity. This may indicate that CART is a strong antioxidant. To further confirm this feature, we also investigated the antioxidant efficacy of CART in HEK293, non-neuronal cells. As shown in Figure 5B, a similar result was obtained. AMVN induced cisPA fluorescence (Control) decay; however, CART treatments (from 1 nM to 20 nM) prevented the fluorescence decrease in a dose-dependent manner, and 20 nM of CART in the reaction almost completely (∼90%) prevented peroxidation. Based on these results, we propose that CART is an antioxidant that efficiently inhibits AMVN-induced oxidation of cisPA.

### Antioxidant role of CART *in vivo*

As an antioxidant CART may have clinical significances, thus we examined the antioxidant outcome of CART peptide *in vivo*. Importantly, PD and its mouse model are dopamine neuron degenerative disorders, in which mitochondrial (especially complex I) dysfunction and oxidative stress have been strongly implicated [29], [34], [35]. The neurotoxin, 1-methyl-4-phenyl-1,2,3,6-tetrahydropyridine (MPTP) mainly impairs complex I, produces oxidative damage in the substantia nigra, results in dopamine neuronal degeneration and parkinsonian symptoms in several species including human, non-human primate and mouse [28], [29], [36]. Thus, we investigated the effect of CART on tyrosine hydroxylase (TH) labeled neurons in the substantia nigra pars compacta (SN-PC) following administration of MPTP to C57BL/6J mice [37], [38]. In addition, motor behavioral tests on the animals were also performed.

**Figure 6:**
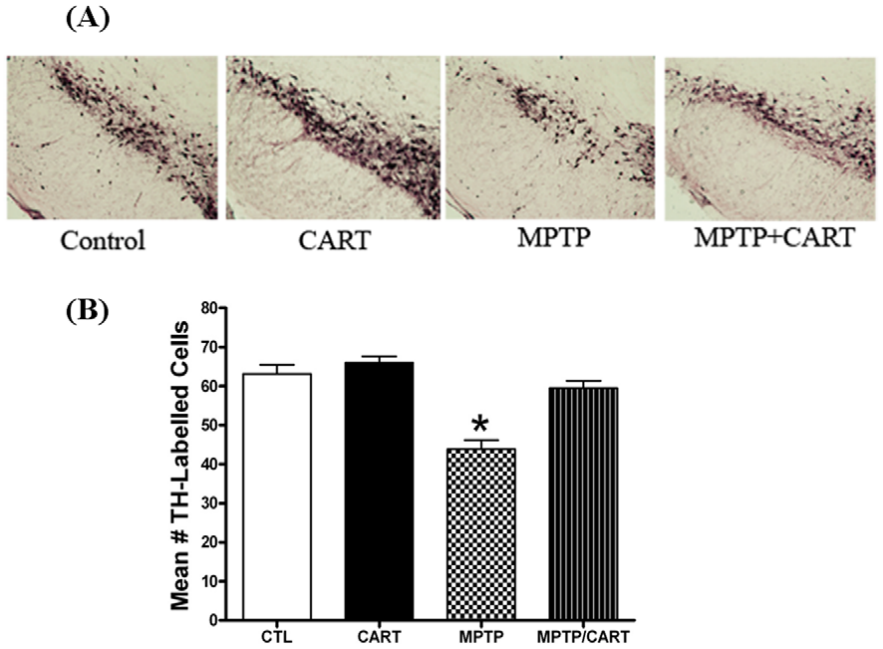
Effect of CART on the number of TH-labeled neuronal cells in the SN-PC. **A.**) Example of TH-immunolabeled neurons within the SN-PC in all four groups: CTL: control group (vehicle + vehicle); CART (CART + vehicle); MPTP (vehicle + MPTP); MPTP/CART (CART + MPTP). There is a decrease in the number of TH-labeled cells in the MPTP only group compared to all other groups. Administration of CART reversed the MPTP-induced decrease in TH-labeled cells. **B.**) CART peptide (50 ng/mouse, IP) or vehicle (0.1 ml/10 grams, IP) was injected one day prior to the start of sub-acute administration of either MPTP (7 mg/kg/d) or vehicle for 7 days. On days 1–7, CART or vehicle were injected 30 minutes prior to administration of either MPTP or vehicle. On day 8, mice were perfused with fixative and the SN-PC cut and processed for TH immunolabeling. CART pretreatment blocked the MPTP-induced decrease in the number of TH labeled cells/section of the SN-PC. *, *p*<0.05 compared to all other groups.

The results of TH immunolabeling in the SN-PC for each group are summarized in Figure 6. A two-way ANOVA (analysis of variance) revealed significant differences between groups (Figure 6A and 6B, *P*<0.0001). Seven days after the first injection of the neurotoxin, post-hoc analysis revealed there was a significant decrease (31%) in the mean number of TH-immunolabeled neurons/section in the SN-PC in the sub-acutely MPTP-treated group compared to the vehicle-treated group (CTL) (Figure 6, *P*<0.001). This decrease in TH-labeling was similar to previous reports on the relative optical density of the SN-PC following sub-acute MPTP exposure [37], [38]. Interestingly, the decrease in TH-labeled neurons in the MPTP group was completely blocked by pretreatment with intraperitoneal injection (50 ng/mouse) of CART peptide (Figures 6A and 6B). These *in vivo* data strongly support our *in vitro* findings, indicating CART also has antioxidant and neuroprotective roles *in vivo*.

**Figure 7:**
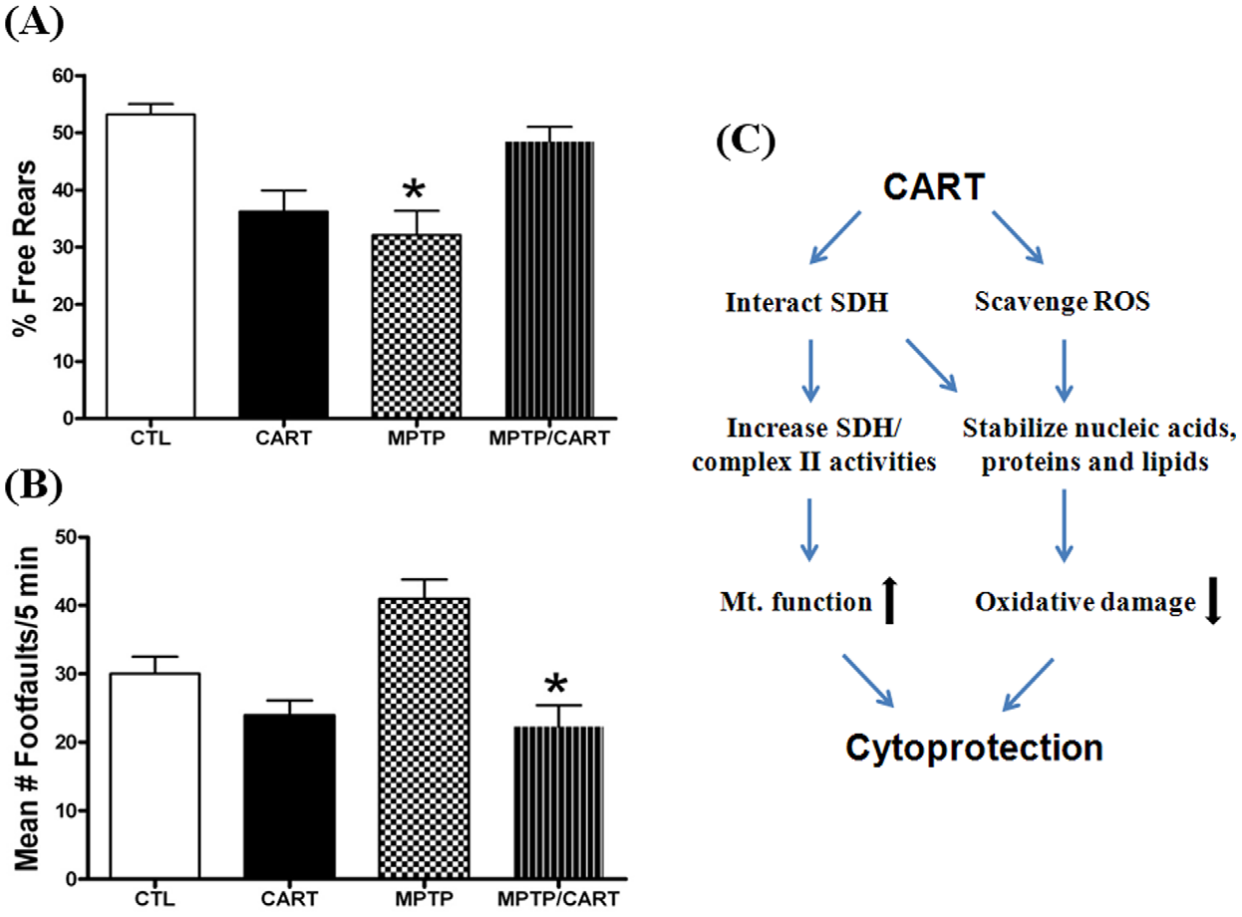
Effect of CART on motor behavior. (**A**) Percent free rears. The number of free versus wall-assisted rears was counted over a 5-minute period on day 8 after the last of the MPTP/CART/vehicle injections and the results presented as percent free rears. For the Control (CTL) group, there was nearly a 50/50 split in terms of the number of free versus wall-assisted rears. There was a decrease in the percent free rears for the CART only group, which was equivalent to that seen in the MPTP treated group. CART pretreatment reversed the MPTP-induced decrease in free rears to the levels seen in the control group. *, *p*<0.05 compared to the CTL and MPTP/CART groups. (**B**) Number of foot-faults. The number of foot-faults during a 5-minute period was determined on day 8 after the last of the MPTP/CART/vehicle injections. CART pretreatment, then followed by MPTP, resulted in a significant decrease in the number of foot-faults compared to the MPTP only group. *, *p*<0.05 compared to the MPTP group. (**C**) Schematic of mechanisms of CART action. Based on our data, CART peptide functions through at least two different and associated pathways, interaction with mitochondrial SDH and directly scavenging oxidants, ultimately preserving cells/neurons.

To determine the effect of CART against MPTP induction on mice behavior, we used the rearing behavioral test that counts the number of times the mouse rears in a cylinder over a 5 minute period and whether the mouse uses the wall to rear or rears free in the middle of the cylinder. For the controls, there is typically a 50/50 split between the percent free versus wall assisted rears [39]. In the MPTP group, with loss of dopamine there is a decrease in the number of free rears, meaning the mouse uses the wall more often to assist in rearing. A two-way ANOVA revealed significant differences between groups (Figure 7A, *P*<0.003). CART was able to reverse the MPTP-induced decrease in the percentage of free rears effects due to MPTP alone (Figure 7A, *P*<0.01). This reversal was associated with the recovery in TH-immunoreactive cells in the SN-PC (Fig. 6). In contrast, wild-type mice treated with CART showed a possible side-effect in this behavioral assay (Figure 7A), indicating normal animals that already have endogenous CART do not need additional CART, which may adversely affect their locomotor behavior. To this point, we propose the use of CART as a therapeutic agent only for PD patients, not for healthy individuals.

To further investigate the alterations in motor behavior, we used a foot-fault test to measure the number of times there was a slip of the paw (either forepaw or hindpaw) through this parallel bar apparatus [39]. When the paw touches the metal floor beneath the parallel bars, the number of such faults is counted. A two-way ANOVA revealed significant differences between groups (Figure 7B, *P*<0.016). The results demonstrate that with MPTP, there was an increase in the number of foot-faults and that CART was able to block this affect (*P*<0.02 between the MPTP and MPTP/CART groups, Figure 7B). This behavioral task further confirmed that CART is protective in this PD mouse model.

## Discussion

In the present study we further demonstrated that CART peptide could enter mitochondria and has an important primary function as an antioxidant. Because of the importance of cellular mitochondria, these findings have shed new light on the molecular mechanisms of CART and its therapeutic possibility for some mitochondria-related diseases, especially neurodegenerative disorders.

### General cellular protection of CART against oxidants

Oxidative stress occurs as a result of an imbalance between the production of ROS and the cell's capacity to neutralize them through its intrinsic antioxidant defenses. Oxidative damage accumulates in normal aging and is involved in the pathogenesis of several neurodegenerative diseases, diabetes, stroke, and cancer [34], [40], [41]. Mitochondria are thought to play a crucial role in the aging process and disease development not only due to their role as the main intracellular generators of ROS, but also because they are targets of ROS attack. An increased production of ROS or a poor antioxidant defense network can lead to progressive cell damage with a decline in physiological function. Consistent with previous reports, our results also revealed that excessive ROS (H_2_O_2_) can damage cellular protein, lipids and DNA, especially mtDNA. Further, our study is the first report that peptide CART significantly reduces the oxidative damage of mtDNA, cellular proteins and lipids in human cells, and directly scavenges ROS. Interestingly, CART's role against oxidative stress is likely stronger than many known antioxidants such as mitoQ, for example, 2 nM of CART were similar to 20 nM of mitoQ in cultured cells [33], [42]. Other antioxidants, such as vitamin C [43], and melatonin [44], [45], were also well studied at pharmacological levels, the doses used in these studies were however mostly higher or much higher than what we used in this CART study.

In addition, CART reduces the neurotoxin/oxidative stress-induced decrease in TH-labeled neurons present in an *in vivo* model of PD, in which oxidative stress is strongly involved, indicating that CART is a potential antioxidant and it may be a hopeful therapeutic agent for some human diseases.

Our previous study showed that CART significantly improved neuronal survival in primary cortical neuronal cells cultured under OGD condition, an *in vitro* ischemia model in which ROS plays a major role [22]. Mitochondiral complex II is thought to be an essential player in hypoxic ROS production [22], [30], [46]. These results are consistent with recent reports [47]–[49] that CART peptide promotes the survival of neuronal cells, including hippocampus and enteric neurons. Interestingly, a neuroprotective role of estrogen is also mediated by CART [48]. Taken together, we propose that CART has a general cellular protective role in mammalian and human cells, at least in part, through its mitochondrial mechanisms, particularly via anti-oxidative function.

### CART as an antioxidant hormone and the mechanisms of its action

Studies have shown that neuroendocrine peptide CART is associated with multiple physiological processes, for instance, feeding, body weight control, and response to drug abuse [6], [7], [9]. As described above, CART is not only highly expressed in the hypothalamus, but also exists in the circulatory system and has a diurnal rhythm [19]. Since these features are similar to many hormones, we considered that CART could be a novel peptide hormone. To date, a physiological hormone with primary antioxidant properties has not been reported. Interestingly, a low dose of CART peptide displayed a strong antioxidant activity (Figure 5). These data are consistent with the idea that CART functions as an antioxidant, which has been defined as “any substance that, when present at low concentrations compared to those of an oxidizable substrate, significantly delays or prevents oxidation of that substrate” [50]. Hence, CART should be considered as a novel antioxidant peptide or antioxidant hormone (AOH).

CART contains three disulfide bridges in its C-terminal part; that seems to form a new unique structure not found in any other proteins [51]. Further, we recently did a database search to find other peptides of similar molecular weight, cysteine numbers and disulfide bonds as CART. However, we were unsuccessful finding such peptides. To date, CART appears to be a new type of peptide. A typical antioxidant such as glutathione, a low molecular weight thiol-containing compound in living cells, should have a reduced form (GSH), which can converted to oxidized form (GSSH) in cells, by acting as a reducing agent for hydroperoxides and free radicals, thus protecting the cells against oxidative damage. Similarly, CART has conformational changes between 6 cysteines (S–H) and 3 disulfides (S–S) bonds (Figure S3). This combined with the mitochondrial localization (Figure S6, and Figures 3 and 4), classifies CART as an AOH, Anti-Oxidative peptide Hormone. The molecular structure also indicates that CART belongs to the family of small thiols. A primary role of small thiols may be one of defense systems against oxidants and other deleterious compounds [52]; our finding regarding the antioxidative properties of CART is consistent with this idea. Therefore we consider the antioxidant role of CART as its primary function.

In the current study we focused primarily on CART's active molecule, CART55-102. While we have not studied other CART fragments, our understanding of CART structure suggests that the antioxidant activity is conferred by the unique motif of 6 cysteine/3 disulfides. Thus, we expect that, CART 1–102 and CART 62–102 would also have the antioxidative activity. However, CART1-27 as the leader sequence does not contain the motif and may thereby not have the antioxidant property. More in depth characterization will be needed to answer these questions unequivocally.

We demonstrated the direct antioxidant property of CART. However, since we previously showed that CART has a transcriptional role in cultured mammalian and human cells [53], we cannot exclude gene-protein expression and signal transduction cascade mechanisms in whole cell and *in vivo*. Other studies also demonstrated that CART could increase brain-derived neurotrophic factor (BDNF) and uncoupling protein UCP-1 expression [47], [54]. As a transcriptional regulator, CART may stimulate other antioxidant gene's expression. This needs further investigation. Our *in vivo* study showed that CART dramatically reduced the decrease in TH-neuronal labeling induced by MPTP. Our data also suggest that the near reversal of TH-labeling in the SN-PC was associated with recovery of motor function, indicating the possibility of future clinical use of CART as a novel antioxidant peptide or hormone.

Interestingly, *in situ* hybridization studies combined with SDH histochemistry and image analysis show that CART mRNA and SDH co-localize in cerebral cortex cells [12], in which SDH is an active metabolism enzyme marker. Using live HEK culture cells, primary cultured neurons and MitoTracker Red, a mitochondrion-selective dye, we first demonstrated that CART preferentially localized into mitochondria. Furthermore, in primary cultured neuronal cells and mouse brain section (Figures 3 and 4), using CART specific antibodies we examined in more detail the location of the CART peptide, and found that native CART is also found in mitochondria, particularly along the outer and inner mitochondrial membranes. This is an important anatomical aspect of CART's action related to mitochondrial stimulation and protection. In contrast, we have shown that presynaptic localization of other peptides (such as the delta opioid receptor, vesicular glutamate transporters 1 and 2) do not cluster around the mitochondria when determined by the same approach [55]–[57]. The specificity of CART localization is clearly demonstrated in the current study, where we show that in unlabeled nerve terminals, there is no labeling of the mitochondria. Therefore, we conclude that CART labeling of the mitochondria in the nerve terminal is specific and it is different from other peptides.

As shown in the published *CART* knockout papers [58]–[60] the *CART* KO mice get obese easily, which combined to our data suggests a direct link between obesity and oxidative stress. However further work with *CART* KO mice including study of their oxidative changes and status of antioxidants will be required to fully understand these observations.

### CART as a potential therapeutic target for Parkinson's disease and other oxidative stress-related diseases

Mitochondria are a common organelle in most mammalian cells, where they supply the majority of the cell's energy. Peptide CART appears to be an endogenous stimulator that boosts mitochondrial function through interacting with SDHB [22]. In the current study, we show that CART can directly scavenge ROS and exert a general cellular protection role against oxidative stress *in vitro*. These observations indicate that CART may be a potential agent for treatment of some diseases in which oxidative stress is involved. For example, since PD is associated with reduced respiratory capacity (as a consequence of primary complex I deficits), increased electron leakage and oxidative stress, feeding electrons into ETC via complex II may be protective in this disease. In addition, CART is closely connected to dopaminergic neurons both anatomically and physiologically [15], [61]–[63]. Dopaminergic neurons are thought to be particularly vulnerable to oxidative stress because they contain dopamine. Thus we chose a common PD animal model to examine the possibility of CART as a therapeutic antioxidant. A previous report indicates that CART readily passes through the BBB (blood–brain barrier) after intravenous injection [64]. However, in the current study we used for the first time intraperitoneal injection of CART. Not surprisingly, our observations from the reduction in the loss of TH-labeled neurons to motor behavioral improvements in the MPTP mouse model of PD indicate that CART can enter the brain and exert a neuroprotective role *in vivo*. This further suggests that CART may be a potential therapeutic agent for PD patients. Clearly this concept will require further validation and additional animal model studies including nonhuman primates before we can completely understand CART role and function with regard to PD. It is encouraged that some patients with neurodegenerative diseases, such as dementia with Lewy bodies [10] have shown a reduced level of CART in their cerebrospinal fluid, indicating the clinical importance of this peptide and it may be a biomarker or therapeutic target for such disease.

Based on our observations, the possible mechanisms for peptide CART are illustrated in Figure 7C. Indeed, a main feature of age- related diseases (such as PD) is oxidative stress, therefore CART acts as an antioxidant to scavenge ROS, and preserves cellular ATP, eventually slows the development of such diseases.

Interestingly, some mitochondrial-oxidative stress-related diseases, such as Huntington's disease [65] and neuroendocrine malignancy [66], exhibit a significant increase in CART peptide levels. The underlying mechanism is unclear, but it may reflect a physiological or pathophysiological response of CART as an endogenous antioxidant peptide/hormone to pathological oxidative damage, rather than a biomarker for the corresponding disease. We interpret this as an adaptive response to the oxidative stress, and postulate it as a new basic molecular mechanism of increased defense or tolerance to cellular oxidative stress. This antioxidative adaptation is an important part of the body defense system, in some cases, it happens not just locally, but also in the circulatory system [67]. Alternatively, the adaptive changes may be the key to treatment for some stress-related disorders [67].

Finally, in the present study, we discovered a new function of CART, its antioxidant activity via which CART protects cellular lipids, protein and mitochondrial DNA against oxidant stress which suggest a general cytoprotective role for CART. A better understanding of the mechanisms through which the antioxidant peptide CART provides neuroprotection may uncover a novel avenue for Parkinson's disease and other neurodegenerative disease therapeutics.

## Methods

### Plasmids and CART fusion proteins

CART (Residues 55–102) [5], [22] and Enhanced GFP (EGFP) cDNAs were amplified by PCR and sequenced to confirm sequence integrity and inserted into the pTAT-2.1 vector (provided by Dr. Steven Dowdy at UCSD). pTAT-2.1, pTATEGFP and pTATEGFP-CART were transformed into *E. coli* BL21(DE3), respectively. The expressions of HisTAT and HisTAT fusion proteins were induced by 0.1 mM *iso*-prppyl-1-thio-β-d-galactopyranoside (IPTG) and were purified by Ni-NTA column (Qiagen) according to the manufacturer's recommended protocol and dialyzed against PBS overnight. The protein concentration was measured by the Bradford method [22]. The bands in the SDS-polyacrylamide gel electrophoresis (PAGE) stained with Coomassie blue or Western blot analyzed by specific antibody indicated the products were correct.

### Cysteine labeling assay

Reactions were carried out with the purified fusion proteins, TATEGFP 20 µg or TATEGFP 40 µg in 10 mM HEPES and 50 mM NaCl (pH 7.0) and supplemented with 0, 20, 100 mM of DTT. All buffers in their bottles and reaction tubes were flushed with N_2_. After incubation for >5 hr at 30°C to reach equilibrium, reactions were quenched with the addition of cold trichloroacetic acid (TCA) to 25% and precipitated on ice for >1 hr. The pellet was collected by centrifugation at 13,000 g, washed with acetone and resolubilized in 1% SDS, 200 mM Na_2_HPO_4_ (pH 7.0) supplemented with 20 mM 4-acetamido-4′-maleimidystilbene-2,2′-disulfonic acid (AMS; MW, 624.33 Da; Invitrogen). The labeling reaction proceeded for 10 min at room temperature and was directly loaded onto a 10% SDS-PAGE. Protein was visualized by Coomassie staining and was imaged.

### Cell line cultures and primary cultures

HEK293 cells were obtained from the American Type Culture Collection (ATCC) maintained in DMEM supplemented with 10% FBS as previously described [22], [68]. Normal and Epstein Barr virus transformed B-lymphocytes were obtained from ATCC and cultured at ∼5×10^5^/mL in RPMI-1640 containing 10% FBS, penicillin (100 units/ml) and streptomycine (100 µg/ml) in 75-cm^2^ tissue culture flasks at 37°C, 5% CO_2_, and 90% relative humidity, and fed periodically by replacement of one third of the medium. Human SH-SY5Y neuroblastoma cells were also obtained from ATCC and cultured under standard conditions in DMEM supplemented with 2 mM L-glutamine and 10% FBS. Cultures of cortical and hippocampal neurons were prepared as previously described [22]. Cellular mitochondria were isolated and mitochondrial complex II activity assay, MTT assay and ATP assay were performed as described previously [22]. All animal related works were approved by our Institutional Animal Care and Use Committees (IACUCs) and followed the NIH *Guide for the Care and Use of Laboratory Animals* (NIH Publications No. 80–23, revised 1978). The approved IACUC numbers for our studies are Oregon Health and Science University 0725, Portland VA Medical Center 3310 and 3705.

### Treatment of cells with CART and H_2_O_2_

HEK293 cells 1×10^6^ or lymphocytes 1.5×10^6^ were plated in duplicate in 60-mm dishes 24–28 hr before treatment. H_2_O_2_ (30%; Fisher) was diluted into phosphate-buffered saline (PBS) and the concentration was determined by absorbance at 240 nm as described [69]. Cultures were pretreated with CART (final concentration for fusion proteins were 5 µg/ml) for 40 min and then exposed to 100 µM H_2_O_2_ (unless otherwise indicated) for either 1 or 2 hr at 37°C in serum-free medium. Non-treatment cells were mock-treated with serum-free medium alone. Cells were washed once with PBS, harvested immediately as described below.

### MtDNA damage assay with semi-quantitative long template PCR

This method detects any DNA lesions including oxidative damage capable of stopping a thermostable polymerase on the DNA template. Hence when using equal amounts of DNA template, increased DNA damages result in decreased PCR amplification of the target sequence. HEK cells and lymphocytes were treated by CART fusions following 100 µM of H_2_O_2_ for 1 h and the total DNA was isolated by the QIAamp DNA isolation kit (Qiagen) as described by the manufacturer. DNA isolation by this method has been shown to be suitable for long PCR, including mtDNA PCR [25]. Isolated DNA (100 ng) was used for PCR amplification of mitochondrial DNA. PCR was performed in a GeneAmp PCR 9700 with the Expand 20 kb PCR kit (Roche) and specific human mitochondrial primers [25] (hmtDNA forward, 5′-TGAGGCCAAATATCATTCTGAGGGGC-3′ and hmtDNA reverse, 5′-TTTCATCATGCGGAGATGTTGGATGG-3′). Briefly, an initial denaturation for 2 min at 92°C followed by 30 cycles of 93°C denaturation for 15 sec, 65°C annealing for 15 sec and 68°C primer extension for 12 min. A final extension at 72°C was performed for 10 min at the completion of the profile. Amplified products (∼16.2 kb) were loaded to a 0.8% agarose gel (with ethidium bromide) and subjected to electrophoresis, the bands were photographed and quantitated with BIO-RAD imaging system.

### Measurement of cellular protein oxidation

To determine the extent of H_2_O_2_-induced protein oxidation, Western analysis was performed using the Oxyblot Protein Oxidation Detection Kit (Chemicon). Near confluent monolayer HEK293 cells pretreated with CART peptide or fusion proteins were treated by addition of H_2_O_2_ to the medium for 2 h and washed with PBS. The cells were scraped into lysis buffer, collected in microcentrifuge tubes, and lysed by sonication. Cell lysates were centrifuged (5000×*g*, 1 min), and the resulting supernatants were collected and the protein concentrations were determined as above. A portion of each sample (15 µg protein) was added to an Eppendorf tube along with 12% sodium dodecyl sulfate (SDS, 5 µl) and dinitrophenylhydrazine (DNP) (10 µl) for 15 min to derivatize oxidized protein residues. The derivatized samples (15 µg protein/lane) were electrophoresed on a 10% SDS–PAGE gel. The derivatized proteins were transferred to nitrocellulose and relative amounts of oxidized protein were determined by Western blot analysis using the primary antibody rabbit anti-DNP and the secondary antibody goat anti-rabbit IgG (horseradish peroxidase conjugated).

### Lipid peroxidation detection

Lipid peroxidation was measured using an LPO586 assay kit (Oxis, Inc., Portland, OR), according to the manufacturer's protocol. Briefly, Lymphocytes were treated and lysed by sonication. A 200-µl sample of each treatment was added to 650 µl of 1∶3 dilution of 10.3 mM *N*-methyl-2-phenylindole in acetonitrile and methanol in a test tube. After mixing by vortex, 150 µl of 15.4 M methanesulforic acid was added. Samples were incubated at 45°C for 60 min and then centrifuged. The supernatants were transferred to cuvettes and absorbance was measured at 586 nm. The 4-HNE diethylacetal standards were diluted in water. The blanks and standards were incubated concurrently with assay samples. Total levels of the two compounds 4-HNE and MDA were measured.

### Cell viability assay

HEK293 cells were treated as described above and then incubated with 3-(4,5-dimethylthiazol-2-yl)-2,5-diphenyl tetrazolium bromide (MTT, Sigma) at a final concentration of 2.0 µg/ml for 3 hr, lysed (20% SDS/50% dimethylformamide), and measured at an absorbance of 570 nm. Absorbance values were converted to MTT reduction, and cell viability was expressed as percent control [22].

### ATP production assay

To examine cellular mitochondrial function, we measured the cellular ATP production by using a sensitive and stable CellTiter-Glo® Luminescent Assay kit (Promega). Briefly, lymphocytes were pretreated with TATEGFP or TATEGFP-CART following addition of H_2_O_2_ to the medium, and then 2×10^4^/well were cultured for 2 h in a 96-well plate in the RPMI-1640. Equal volume of CellTiter-Glo reagent was added to each well for 15 min and the luminescence was detected by a Viatras luminometer.

### Mitochondria localization of CART in cultured cells

**(1)** In live HEK cells. Cells in 6-well plates were treated with 8 µg/ml of TATEGFP-CART or TATEGFP, respectively. 24 h after treatment the cells were incubated with 50 nM of MitoTracker Red CMXRos (Invitrogen) according to the manufacturer's instructions. The cells were then washed with PBS and fresh medium was added to the cells. Cells were subjected to a Leica TCS SP confocal laser scanning microscopy (Leica, Wetzlar, Germany). Images were visualized, digitally recorded and then colorized using Adobe Photoshop. **(2)** In primary neuron cells. The cortex and hippocampus of C57BL/6J mouse were dissected and reserved for primary culture as described previously [22], [70]. Neuronal cells were cultured on cover slides for one week, and incubated with 20 nM of MitoTracker Red CMXRos for 1 hr. The neuron cells were fixed with 4% paraformaldehyde and incubated with CART specific antibody (Santa Cruz, 1∶100) following a biotinylated secondary antibody (1∶300). Then cells were incubated with the ABC solution (1∶500 dilution, Vector Laboratories) and treated with tyramide-conjugated fluorescent dye Alexa 488 (green) (Invitrogen) and mounted with prolong gold then photographed using a fluorescence microscope at 100× magnification.

### Electron microscopic immunohistochemistry

Three naïve C57BL/6J mice (Jackson Labs, Bar Harbor, ME) were anesthetized with isoflurane, the chest cavity opened and perfused transcardially with 3 mls of heparin (1000 units/ml) in 0.1 M PBS (pH 7.3), followed immediately by 35 ml of 1% glutaraldehdye/0.5% paraformaldehdye/0.1% picric acid in 0.1 M PBS (pH 7.3). The brains were fixed overnight and then cut on a vibratome at 60 µm. The tissue was incubated in a microwave (Pelco BioWave, Ted Pella, Inc, CA) for 5 minutes, 550 watts (W), at 35 degrees C (all the remaining steps occurred at this temperature without vacuum unless specified) in 10 mM sodium citrate, pH 6.0, rinsed in 0.1 M PBS for 2×1 min at 150 W, exposed to 3% hydrogen peroxide at 150 W for 1 min, rinsed in PBS at 150 W for 2×1 min, exposed to 0.5% Triton X-100 for 5 min, 550 W with the vacuum on, washed in phosphate buffer for 2×1 min at 200 W with the vacuum off, then exposed to the primary antibody (rabbit polyclonal, 1∶100; Phoenix Pharmaceuticals, Inc, CA) for 12 minutes at 200 W, 4 times using the following cycle: 2 min on, 2 min off, 2 min on, 5 min off, all on a continuous vacuum [56]. The tissue was then rinsed in phosphate buffer, 2×1 min, at 150 W, then exposed to the secondary antibody (biotinylated goat anti-rabbit, 1∶500; Vector Labs, CA) for 15 minutes at 200 W for two cycles of the following: 4 min on, 3 min off, 4 min on, 5 min off, all on a continuous vacuum. The tissue was then rinsed in PBS, 2×1 min, at 150 W, then exposed to ABC (Vector Elite Kit, 1 µl/ml of solution A and B in phosphate buffer) for 11 minutes at 150 W, under vacuum, using the following cycle: 4 min on, 3 min off, 4 min on. The tissue was then rinsed in phosphate buffer, 2×1 min, at 150 W and exposed to diaminobenzidene (0.5 µg/ml+1.5% hydrogen peroxide) for up to 10 minutes. The sections were then processed for embedding in epoxy resins as previously described [37], [56]. Thin sections were cut on an ultramicrotome (Leica Ultracut) and then viewed and photographed at ×40,000 on a JEOL 1400 electron microscope. Digital images were captured using an AMT (Danvers, MA) 2 K×2 K digital camera.

### Radical scavenging assays

Mitochondria of cultured cells were isolated and measured as previously described [22]. SH-SY5Y mitochondria (50 µg), cisPA (cis-trans-trans-cis-9,11,13,15-octadecatetraenoic acid, Molecular Probe, Eugene, OR) were added into 0.1 M KCl and 50 mM PBS. The final concentration of cisPA was 12 µM. The reaction was started by increasing the sample temperature to 40°C immediately after the addition of AMVN (Cayman Chemical, 1.5 mM, zero time) and CART peptide (CART55-102, Phoenix, final concentration: 1 nM) was added 20 min after the addition of AMVN. The measurements of fluorescence of cisPA were performed on a spectrofluororometer (BIO-TEK) every 5 min for 60 min. To further verify the scavenging role of CART in different cells, HEK cellular mitochondria (50 µg) containing cisPA (6 µM) and different concentrations of CART (0, 1, 10, and 20 nM) were prepared. The reaction was started with addition of AMVN (2 mM) at 40°C for 20 min, incubation was continued at RT for 90 min, and fluorescence intensity was then measured by the spectrophotometer as described above.

### PD mouse model and drug treatment

C57BL/6J mice (males, 10 weeks old, Jackson Labs, Bar Harbor, Maine), were maintained on a 12 hour light/dark cycle with continuous access to food and water. All necessary precautions were taken in handling, injecting and disposal of MPTP and the mouse caging material [36], [37], [71]. The mice were first injected with the CART peptide (CART55-102, Phoenix, 50 ng in normal saline/mouse, IP) or vehicle (normal saline, 0.2 ml/mouse, IP). Then one day later, animals were injected with the CART peptide or vehicle and 30 minutes later, administered MPTP (7 mg/kg, IP) or vehicle, as previously described [39]. This sequence of injections continued for 7 days. The following groups were tested: control (vehicle/vehicle, n = 4), CART (CART/vehicle, n = 4), MPTP (vehicle/MPTP, n = 6), CART/MPTP (n = 7). One day following the last injection, and after the behavioral testing, mice were anesthetized with isoflurane and then perfused with 50 ml of fixative (1% acrolein/2% paraformaldehyde in 0.1 M PBS, pH 7.2) [37], [38].

### Behavioral analysis

**(1)** Rearing Test. One day after the last injection of drug (ie day 8), the mice were placed in the middle of a plastic cylinder (6 cm wide ×15 cm deep, black colored floor) and the number of free or unassisted rears (i.e. mice reared up without the need to support themselves by the wall) and the number of assisted rears (i.e. mice reared up with the need to support themselves by the wall) was counted over a 5 minute period, as previously described [39]. There was no acclimation time prior to testing. The number of free and assisted rears was totaled and the data presented as mean percent of free rears. Between animal testing, the chamber was cleaned with 10% isopropyl alcohol. **(2)** Foot-fault. The number of foot-faults or slips through parallel bars over a 5 minute period was recorded using a parallel rod floor apparatus as previously reported [39] and tested for reliability [72]. The apparatus consists of a 15×15×20-cm clear acrylic plastic box with a removable top. The steel rods are 1.6 mm in diameter, separated from each other by 6 mm. A stainless steel plate is set 1 cm below the rod floor. When the mouse's paw slips through the parallel rods and contacts the metal plate, a circuit is closed and an error recorded by the computer. Slips of the tail or feces through the rods are not recorded [63]. The mice were acclimated to the testing chamber for 5 minutes prior to the recording of the footfaults. Between animal testing, the chamber was cleaned with 10% isopropyl alcohol. The data are presented as the mean number of footfaults/5 minutes.

### Tyrosine hydroxylase (TH) immunolabeling

To determine the mean number of TH immunolabeled neurons within the substantia nigra pars compacta (SN-PC) after the treatments, following the completion of the behavioral experiments, animals were anesthetized with isoflurane and perfused with fixative (see above), washed overnight in PBS, and then cut at 70 µm using a vibratome (Leica Microsystems, Bannockburn, IL) [39]. Sections were serially cut throughout the entire rostral/caudal extent of the substantia nigra. Eight sections covering the full extent of the substantia nigra were incubated in individual wells (48 well plate) at RT for 30 minutes in 1% sodium borohydride (in 0.1 M PBS) and then washed several times (5×10 minutes) in PBS. For antigen retrieval, the sections were incubated in a microwave (Ted Pella, Redding, CA) for 5 minutes in 500 µl of 10 mM sodium citrate (pH 6.0) at 550 Watts, 60°C, with no vacuum. The tissue was washed in PBS and incubated in the cold room (4°C) in blocking solution (5% normal goat serum, 0.5% triton X-100, in PBS) for 1 hour. The blocking solution was then replaced with antibody [mouse anti-TH sera (Diasorin, Stillwater, MN), diluted 1∶20,000 in blocking solution] and incubated overnight in the cold room. The sections were then washed in PBS, followed by RT incubation in biotinylated goat anti-mouse IgG (Vector Labs, Burlingame, CA, diluted 1∶250) for 45 minutes, washed in buffer, and then exposed to avidin-biotin complex (Elite Kite, Vector Labs, diluted per manufacturers instructions). The sections were washed in buffer and exposed to 3,3′ diaminobenzidine hydrochloride (DAB kit, Vector Labs, diluted per manufacturers instructions). Sections were mounted serially on gelatinized slides, left to dry overnight and then coverslipped. Digital photographs were taken of each side of the substantia nigra, the number of TH-labeled neurons counted and the number of TH-positive neurons averaged between the two sides of the brain. The number of TH-labeled neurons per section (averaged between the two sides) of the SN-PC were averaged between the 8 sections/animal and the overall mean number determined for each experimental group. From these tissue section counts, the total number of labeled neurons was re-evaluated using the Abercrombie correction, which accounts for fragmented nuclei within each section and provides an accurate estimate when tissue thickness exceeds soma thickness by more than 50%, which is the case in this study, as previously described [39]. Although this cell counting methodology may have yielded an underestimation of the total number of TH immunolabeled neurons/section in the SN-PC, it is an appropriate approach for measuring the mean number of neurons/section according to recent comparisons of 2D and 3D analyses of brain tissue [73], [74].

### Statistical Analysis

Reported values are means±SEM. Observed mean differences between controls and treated groups were analyzed by Student's *t* test. Where multiple data were compared, an ANOVA was employed before group comparisons were made. For the mean number of TH immunolabeled cells/section and both behavioral measures, the differences between groups were determined using a two-way ANOVA, with significant main effects characterized using the Tukey-Kramer *post-hoc* for comparison of multiple means. A *P*-value of <0.05 was considered to be statistically significant.

## Supporting Information

Figure S1 **Tissue expression of CART peptides determined by Western blot analysis.** Several rat tissues were isolated from adult female rat, 20 µg of protein was run on a peptide gel (Invitrogen) and transferred to a PVDF membrane, then blotted by primary antibody against CART. Two major sizes (around 4∼10 kDa) of CART peptides were strongly detected in hypothalamus and pituitary.
(TIF)

Figure S2 **Purified CART fusion proteins were recognized by CART-antibody.** Fusion proteins were isolated from bacteria and 2 µg of each protein was run on a 10% SDS-PAGE gel and Western blot was performed using specific CART-antibody.
(TIF)

Figure S3 **A structure change of TAT-EGFP-CART under reducing conditions confirmed the fact that original key feature of CART exists in the fusion protein.** A cysteine reactivity assay using a small maleimide reagent (∼500 Da added per free thiol) shows that TAT-EGFP no significant molecular weight changes (A); however TAT-EGFP-CART fusion proteins into a reduced form upon incubation with the reducing agent dithiothreitol (DTT) (B), demonstrating that the redox of CART functions (systeines-disulfides change) in the TAT-EGFP-CART fusion proteins.
(TIF)

Figure S4 **TAT-EGFP-CART prevents mitochondrial dysfunction after oxygen-glucose deprivation in primary cultured cortical neurons.** TAT-EGFP-CART or TAT-EGFP was added at 0.2 nM concentration 30 min prior to 2 hr OGD, and mitochondria extracted at 24 hr after OGD. Complex II activity in mitochondrial extract was measured spectrophotometrically at 595 by 2, 6-dichloroindiphenol (DCIP) reduction after the addition of Coenzyme Q at 3 min.
(TIF)

Figure S5 **Mitochondrial DNAs were amplified by long template PCR.** Total DNA was isolated from differently treated HEK cells and PCR was performed using specific human mitochondrial primers and Expand 20 kb DNA amplification kit. Lane 1, 1 kb ladder, lane 2, HEK cell control (100% band density), lanes 3 and 4, HEK cells treated with H_2_O_2_ for 30 min (91% of control) and 60 min (22% of control).
(TIF)

Figure S6 **Confocal imaging** **of TAT-EGFP-CART in live cells shows CART fusion proteins preferentially localized into mitochondria comparing with TAT-EGFP control.** Cultured HEK293 cells were treated overnight with 8 µg/ml of TAT-EGFP-CART or TAT-EGFP fusion proteins as indicated, and then treated with 50 nM of MitoTracker Red CMXRos for 30 min. Living cells were analyzed by a confocal microscopy. Top row, confocal fluorescence images depict cells treated with TAT-EGFP-CART fusion proteins; and bottom row, confocal fluorescence images depict cells treated with vehicle TAT-EGFP fusion proteins. Cells in panels A and D show GFP (green), cells in panels B and E show mitochondria (red), and panels C and F are merged images (yellow). Pixels containing fluorescence for both GFP and the red MitoTracker appear as yellow in the merged images. Scale bars: 9.95 µm (A–C) and 18.8 µm (D–F).
(TIF)
