# Supplementary figures and images for: CART Peptide Is a Potential Endogenous Antioxidant and Preferentially Localized in Mitochondria
Source: PLoS One. 2012 Jan 3;7(1):e29343. doi: 10.1371/journal.pone.0029343 (PMC3250433; doi:10.1371/journal.pone.0029343)

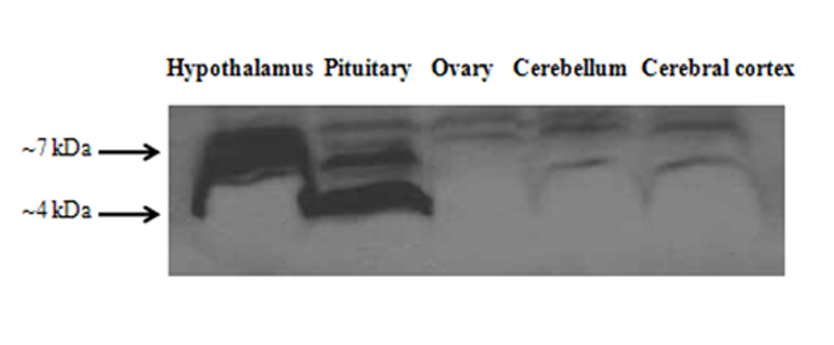

Supplement: Figure S1 — Tissue expression of CART peptides determined by Western blot analysis. Several rat tissues were isolated from adult female rat, 20 µg of protein was run on a peptide gel (Invitrogen) and transferred to a PVDF membrane, then blotted by primary antibody against CART. Two major sizes (around 4∼10 kDa) of CART peptides were strongly detected in hypothalamus and pituitary. (TIF) [file pone.0029343.s001.tif]

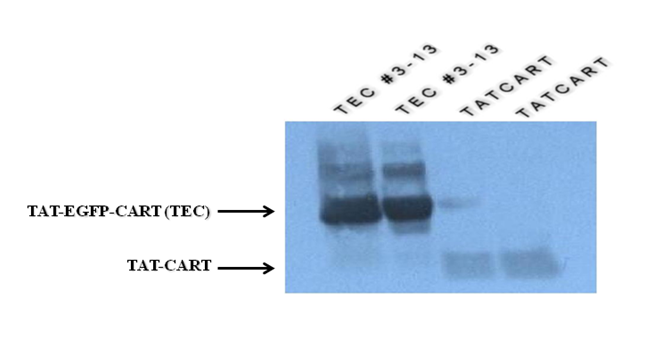

Supplement: Figure S2 — Purified CART fusion proteins were recognized by CART-antibody. Fusion proteins were isolated from bacteria and 2 µg of each protein was run on a 10% SDS-PAGE gel and Western blot was performed using specific CART-antibody. (TIF) [file pone.0029343.s002.tif]

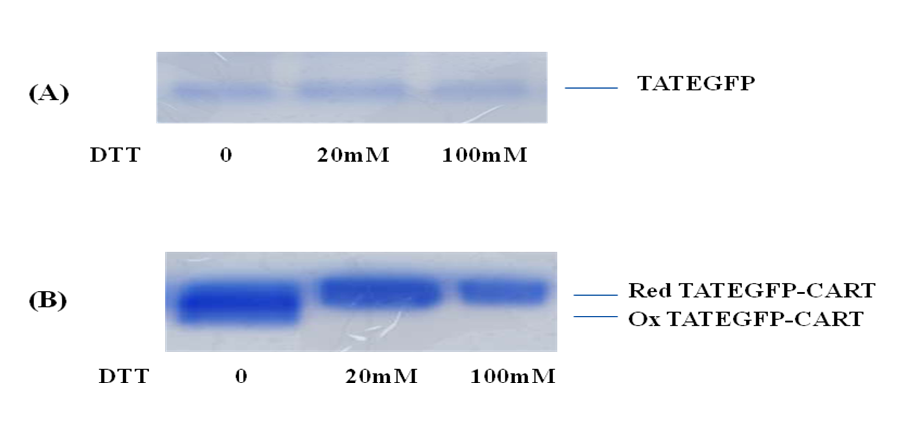

Supplement: Figure S3 — A structure change of TAT-EGFP-CART under reducing conditions confirmed the fact that original key feature of CART exists in the fusion protein. A cysteine reactivity assay using a small maleimide reagent (∼500 Da added per free thiol) shows that TAT-EGFP no significant molecular weight changes (A); however TAT-EGFP-CART fusion proteins into a reduced form upon incubation with the reducing agent dithiothreitol (DTT) (B), demonstrating that the redox of CART functions (systeines-disulfides change) in the TAT-EGFP-CART fusion proteins. (TIF) [file pone.0029343.s003.tif]

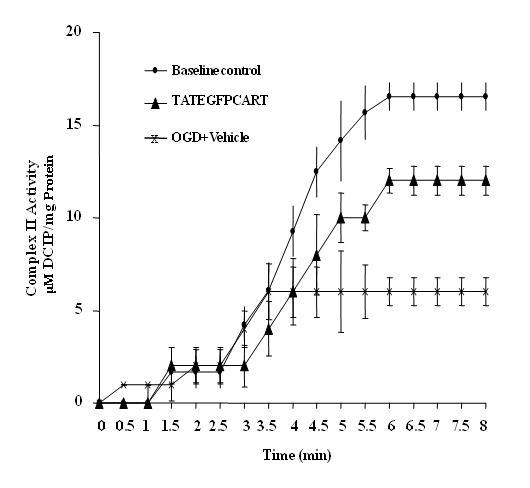

Supplement: Figure S4 — TAT-EGFP-CART prevents mitochondrial dysfunction after oxygen-glucose deprivation in primary cultured cortical neurons. TAT-EGFP-CART or TAT-EGFP was added at 0.2 nM concentration 30 min prior to 2 hr OGD, and mitochondria extracted at 24 hr after OGD. Complex II activity in mitochondrial extract was measured spectrophotometrically at 595 by 2, 6-dichloroindiphenol (DCIP) reduction after the addition of Coenzyme Q at 3 min. (TIF) [file pone.0029343.s004.tif]

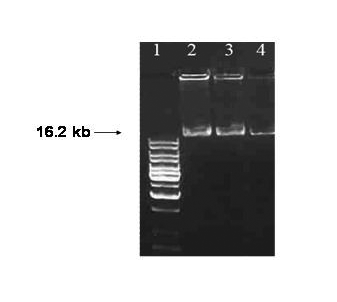

Supplement: Figure S5 — Mitochondrial DNAs were amplified by long template PCR. Total DNA was isolated from differently treated HEK cells and PCR was performed using specific human mitochondrial primers and Expand 20 kb DNA amplification kit. Lane 1, 1 kb ladder, lane 2, HEK cell control (100% band density), lanes 3 and 4, HEK cells treated with H2O2 for 30 min (91% of control) and 60 min (22% of control). (TIF) [file pone.0029343.s005.tif]

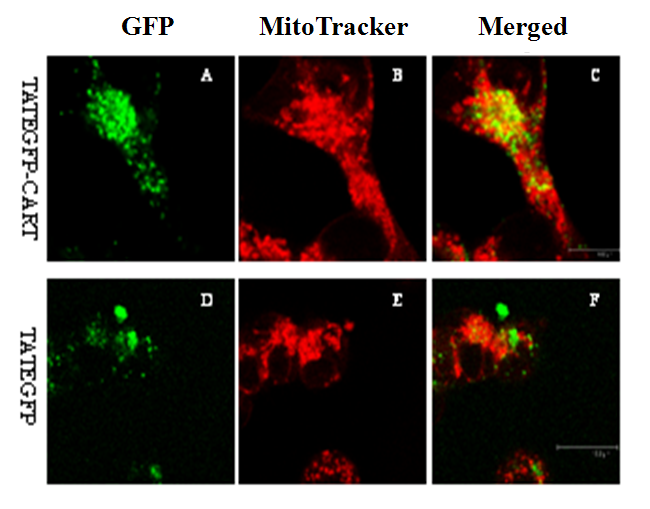

Supplement: Figure S6 — Confocal imaging of TAT-EGFP-CART in live cells shows CART fusion proteins preferentially localized into mitochondria comparing with TAT-EGFP control. Cultured HEK293 cells were treated overnight with 8 µg/ml of TAT-EGFP-CART or TAT-EGFP fusion proteins as indicated, and then treated with 50 nM of MitoTracker Red CMXRos for 30 min. Living cells were analyzed by a confocal microscopy. Top row, confocal fluorescence images depict cells treated with TAT-EGFP-CART fusion proteins; and bottom row, confocal fluorescence images depict cells treated with vehicle TAT-EGFP fusion proteins. Cells in panels A and D show GFP (green), cells in panels B and E show mitochondria (red), and panels C and F are merged images (yellow). Pixels containing fluorescence for both GFP and the red MitoTracker appear as yellow in the merged images. Scale bars: 9.95 µm (A–C) and 18.8 µm (D–F). (TIF) [file pone.0029343.s006.tif]
